# How does stretching exercise of the ankle joint affect balance and gait function in healthy older adults aged 65 to 75 years?: A randomized clinical trial

**DOI:** 10.1097/MD.0000000000046567

**Published:** 2025-12-12

**Authors:** Hyeon Woo Ryoo, Soo-Kyung Bok, Myeong Hyeon Cho, So Young Ahn

**Affiliations:** a Department of Rehabilitation Medicine, Chungnam National University Hospital, Jung-gu, Daejeon, Republic of Korea.

**Keywords:** ankle joint stretching, elderly, gait function, postural stability

## Abstract

**Background:**

This study aimed to examine the effects of ankle joint stretching exercises on gait function in older adults and to clarify their relationship with balance control. Specifically, the study compared the outcomes of simultaneous stretching of the plantar flexors and ankle inverters with those of stretching the plantar flexors alone.

**Methods:**

In this single-blind randomized controlled trial, 40 healthy older adults aged 65 to 75 years (36 women and 4 men; overall mean age, 69.0 ± 2.9 years) were randomized (1:1) to either the experimental group (simultaneous stretching of the plantar flexors and invertors) or the control group (stretching of the plantar flexors only). Each group performed stretching exercises for 30 minutes/day, 5 days/week, for a total of 15 sessions over 3 weeks. The primary outcome was the Ankle dorsiflexion and eversion range of motion (ROM), Berg Balance Scale (BBS) score, Timed Up and Go test (TUG), gait parameter, center of pressure (COP) parameter.

**Results:**

Both groups showed significant improvements in ankle dorsiflexion ROM, BBS, step cadence, and double support time in gait after the intervention. However, only the experimental group showed significant improvements in ankle eversion ROM, mediolateral COP range, step velocity on both sides, and TUG performance. Between-group comparisons revealed that changes in eversion ROM, mediolateral COP range, and TUG performance were significantly greater in the experimental group than in the control group. No significant between-group differences were observed in dorsiflexion ROM, BBS scores, step length, step duration, mean COP velocity, or cadence.

**Conclusion:**

Ankle stretching exercises improved balance and gait function in healthy older adults, with greater effects observed from combined stretching of the plantar flexors and invertors. These benefits are likely due to enhanced postural control and increased ankle flexibility.

## 1. Introduction

In modern society, the population of individuals aged 65 and above is rapidly increasing. Falls represent a significant public health concern, primarily occurring during ambulation in older adults. Fall-related injuries are a major cause of mortality and morbidity in older adults. Additionally, fear of falling may restrict both physical and social activities, potentially resulting in a decline in quality of life. Most falls in older adults are associated with multiple risk factors, including muscle weakness, impaired balance control, gait disorders, visual impairments, medication use, and environmental factors.^[1,2]^ Balance control is a complex phenomenon influenced by various neurological and musculoskeletal factors. Balance is maintained through the sensory detection of body movements, integration of sensory-motor information within the central nervous system, and the execution of appropriate musculoskeletal responses.^[3]^

Various studies have revealed that specific foot and ankle characteristics play crucial roles in balance and postural control. Ankle muscle response times required to maintain balance are significantly longer in older adults than in younger individuals, and ankle muscle strength notably decreases with age. Furthermore, age-related deterioration of joint proprioception impairs balance control and restricts joint range of motion (ROM), thereby limiting daily activities in older adults. Ankle plantar flexion and dorsiflexion strength, along with ankle joint ROM, are key contributors to balance and gait performance. Notably, reduced ankle dorsiflexion ROM has emerged as an independent predictor of falls.^[4]^ Previous research has highlighted the significance of ankle eversion ROM as a determinant of balance and functional ability in older adults.^[5,6]^

Previous studies have implemented various rehabilitation interventions, including strengthening and stretching exercises, to enhance balance performance.^[7–12]^ Stretching can induce biomechanical and physiological changes in muscles, resulting in adaptive alterations in ROM, sensory perception, and passive torque. Such adaptive changes in the lower extremities, including the calf muscles, may influence postural control strategies and balance performance.^[13]^ Martinez-Jimenez et al^[14]^ reported improved static balance following static stretching of the lower limbs in healthy adults. However, despite numerous studies investigating the relationship between stretching and balance, the effects remain inconsistent, and the underlying mechanisms are not well understood.^[14–16]^ Moreover, a specific exercise protocol that optimally enhances balance and functional ability in older adults has yet to be established.

It is also important to consider whether balance improvements from ankle stretching translate into enhancements in gait performance. Giboin et al^[17]^ noted that the effects of balance training are task-specific, improving performance in trained tasks but not necessarily in untrained tasks. Conversely, improvements in clinical balance scores, gait parameters, Timed Up and Go (TUG) scores, and other functional assessments have been proposed to represent a transfer of training effects from static balance exercises to dynamic gait stability.^[18–20]^ Additionally, the incidence of falls decreases when standing balance improves.^[21]^ Therefore, it remains unclear whether improved standing balance through training, including ankle joint stretching exercises, ultimately leads to enhanced gait performance.

Therefore, this study aimed to investigate the effects of ankle joint stretching exercises on gait function in older adults and to elucidate their association with improved balance control. Additionally, we aimed to identify targetable factors for an optimal exercise program to enhance balance and prevent falls by comparing the effects of simultaneous stretching of the plantar flexors and inverters with those of stretching of the plantar flexors alone.

## 2. Materials and methods

### 2.1. Participants

This study was approved by the Institutional Review Board of Chungnam National University (number: CNUH 2021-12-009) and registered with the Korean Clinical Research Information Service (number: KCT0010688). From December 2021 to March 2023, participants were recruited via a public notice posted on the outpatient and inpatient bulletin boards of the Department of Rehabilitation Medicine at Chungnam National University Hospital. Written informed consent was obtained from all patients who participated in the study. Inclusion criteria for the study were as follows: healthy older adults, aged 65 years or older but <75 years; independent ambulation without issues in lower limb strength and cognitive function. This age range corresponds to the “young-old” category (65–74 years), a classification widely adopted in gerontology and geriatrics research.^[22–24]^ This categorization is clinically relevant because objective studies have shown that measurable declines in balance and gait performance tend to emerge from the mid-60s and become more pronounced by the early 70s, while the burden of multi-morbidity, although already present, remains relatively lower than in adults over 75 years.^[25–28]^ In addition, randomized controlled trials in geriatric populations have also employed this age stratification to investigate functional decline and fall risk, supporting its clinical and methodological relevance.^[29]^ Exclusion criteria were as follows: individuals with a body weight of 100 kg or more; individuals with musculoskeletal or neurological conditions that could affect ambulation; individuals who underwent foot or ankle-related surgical procedures within the past 6 months; and individuals with impaired cognitive function hindering participation in the study.

### 2.2. Sample size

The sample size was calculated using the G*Power 3.1.9.2 program. Based on a previous related study,^[15]^ the significance level (α) was set at 0.05 and the statistical power at 0.8. Consequently, a sample size of 44 was required. Considering a dropout rate of 10%, 49 participants were required. Finally, 44 individuals volunteered to participate in this study.

### 2.3. Randomization and masking

Participants were randomly assigned to either the experimental group (simultaneous stretching of the plantar flexors and invertors) or the control group (stretching of the plantar flexors only) in a 1:1 ratio according to a computer-generated random number table. Group allocation was concealed using sealed, opaque envelopes prepared by an independent researcher. This study was conducted as a single-blind randomized controlled trial; the participants were blinded to each group assignment, whereas the researchers were not blinded due to the nature of the intervention. All outcome assessments were conducted by an independent researcher who was not involved in the intervention and participants were instructed not to disclose their group assignment during assessments.

### 2.4. Procedure & intervention

**Figure 1.**
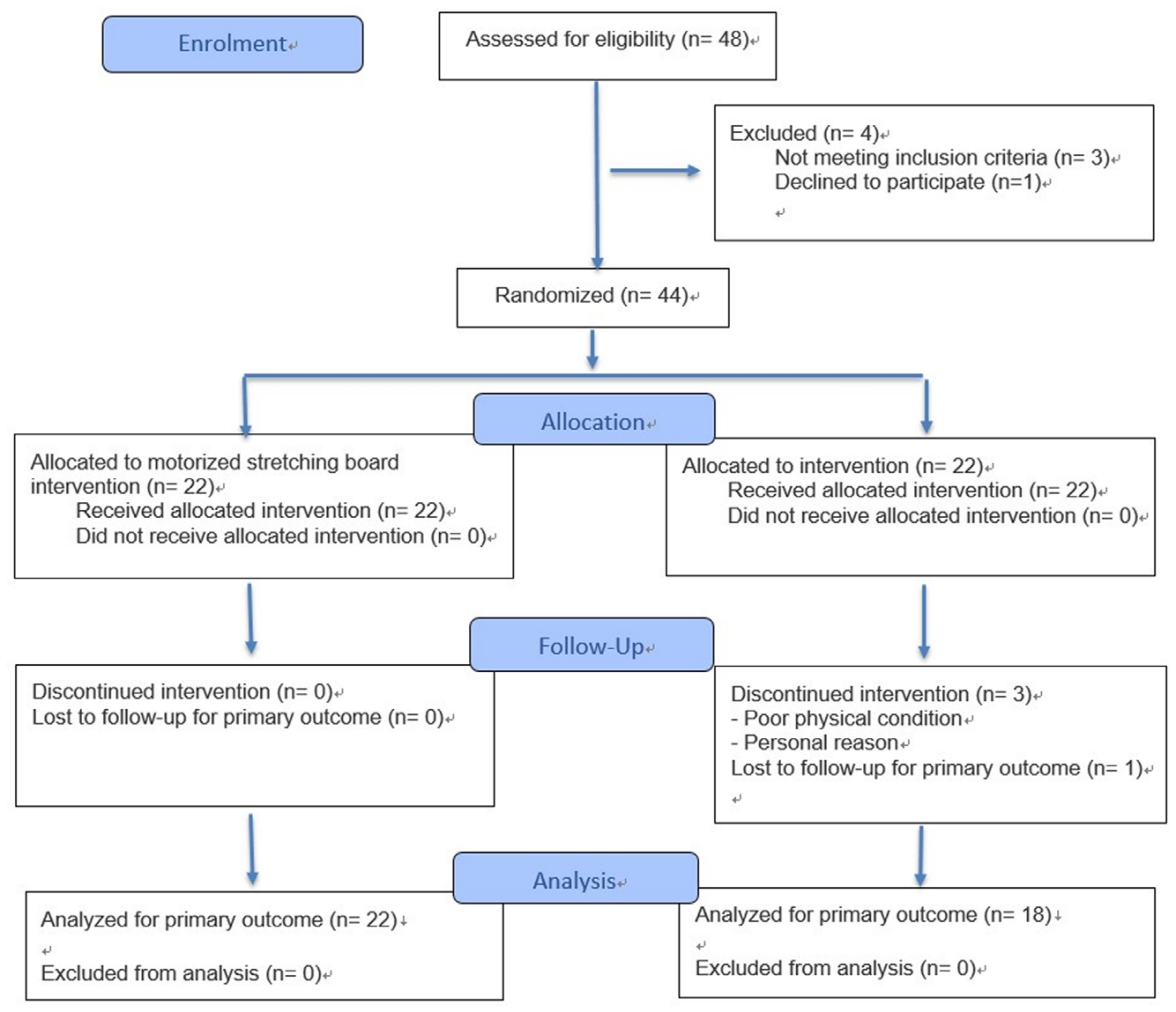
Flow diagram showing the enrollment, randomization, allocation, follow-up, and analysis of study participants.

Before the participants were assigned to groups, their general characteristics (age, sex, height, weight, BMI, and foot size) were recorded. 44 participants were randomly assigned to either the experimental group (simultaneous stretching of the plantar flexors and invertors) or the control group (stretching of the plantar flexors only), with 22 participants per group. During the study, 4 participants in the control group withdrew due to poor physical condition and personal rea-sons; thus, data from 40 participants were analyzed (Fig. 1). Participants with missing data were excluded from the analysis. All interventions were performed by physical therapists with more than 3 years of clinical experience and were conducted at Chungnam National University Hospital. The experimental group used a motorized stretching board, whereas the control group performed wall stretching, a static stretching exercise commonly used in clinical settings. Each group performed stretching exercises for 30 minutes/day, 5 days/week, for a total of 15 sessions over 3 weeks. The frequency and duration of the stretching sessions were based on previous studies demonstrating the effectiveness of ankle plantar flexor stretching exercises.

#### 2.4.1. Wall stretching

**Figure 2.**
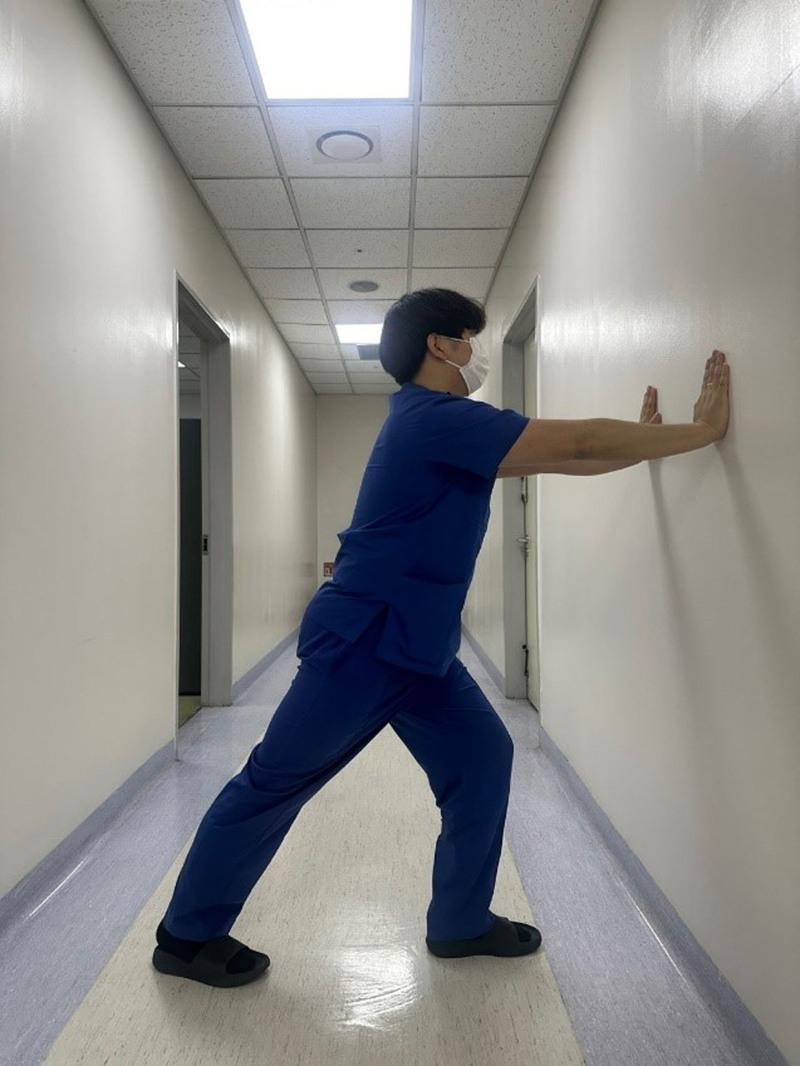
Wall stretching position targeting the plantar flexor muscles, performed with the rear foot in a toe-in posture, the rear leg extended, trunk upright, and heel grounded.

For wall stretching (Fig. 2), participants were asked to stand facing a wall with the lower limb stretched behind and the contralateral leg placed in front. Participants were instructed to keep their trunk upright and flex the knee of the front leg until a stretch was felt in the triceps surae of the back leg, ensuring the heel remained on the ground throughout the stretch. According to a previously published method,^[30]^ participants used a “toe-in” position, with the toes of the back foot pointing toward the heel of the front foot.

#### 2.4.2. A motorized stretching board, “Ankle Stretcher”

**Figure 3.**
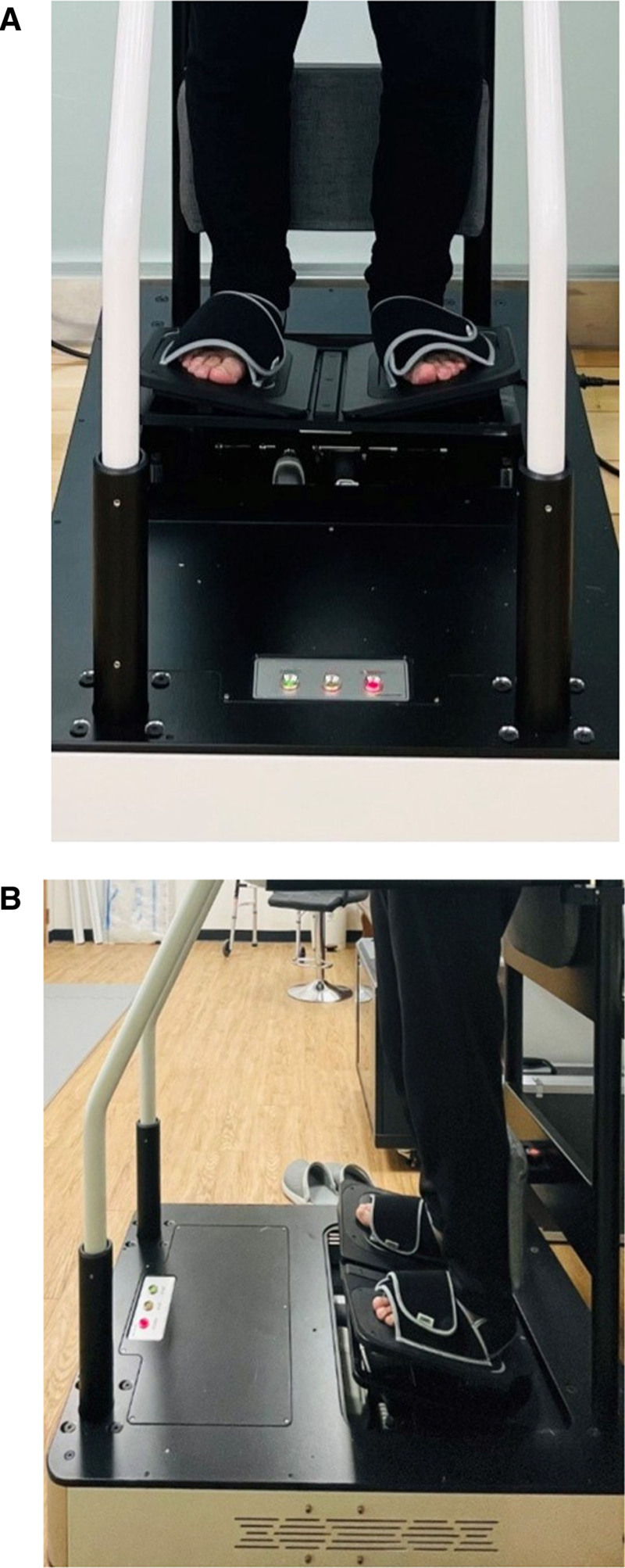
(A) Dorsiflexion and eversion stretching of the ankle joint using a motorized stretching board “Ankle Stretcher.” Frontal view of the Ankle Stretcher device. The participant’s feet are secured with straps. (B) Lateral view of the Ankle Stretcher device. Demonstrating the ankle in the stretched position during training.

In this study, we utilized an Ankle Stretcher (IT CO., Ltd., Gyeongsangbuk-do, Korea) and a motorized stretching board (Fig. 3). The “Ankle Stretcher” comprises 2 footplates, each equipped with a linear inline actuator positioned beneath an actuator controller, an operational tablet PC, and a safety bar. It is a medical rehabilitation device capable of controlling various ankle angles using built-in electric motors. When the user’s weight is applied, the Ankle Stretcher induces dorsiflexion and inversion movements of the ankle, stretching the muscles on the medial and posterior aspect of the calf, as well as the Achilles tendon. The device allows angle adjustment for dorsiflexion from 0 to 30° and inversion from 0 to 25°, along with control over stretching speed, duration, and rest intervals. When the participant stepped onto the Ankle Stretcher without shoes, both feet were secured using fixation straps, and the safety frame was adjusted to prevent falls. The initial dorsiflexion (10°) and eversion (5°) angles were gradually increased until the participant felt a painless stretching sensation in the Achilles tendon and surrounding ankle muscles, and this position was maintained for 30 minutes of training (Fig. 2). During each movement cycle driven by the Ankle Stretcher, dorsiflexion and eversion durations were set to 20 seconds, followed by a 10-second period of comfortable standing in the lowered position. This process was repeated for 30 minutes of training.

### 2.5. Primary outcome variables

Participants were evaluated at 2 time points: prior to the intervention and immediately after completing the 3-week intervention period.

#### 2.5.1. Ankle joint ROM

The passive joint ROM of ankle dorsiflexion was evaluated using a goniometer with high reliability (intraclass correlation coefficient [ICC], 0.68–0.89). The ROM for dorsiflexion was measured as the angle between the line connecting the fibular head and the outer malleolus and the longitudinal axis of the 5th metatarsal bone.^[31]^ The eversion ROM was assessed while the participants were seated. Landmarks were placed on each participant at the midpoint between the malleoli on the anterior aspect of the ankle, the midline of the anterior lower leg using the crest of the tibia as a reference, and the longitudinal midline on the dorsal surface of the second metatarsal. A goniometer was aligned with the landmarks on the ankle and foot, and the participants actively moved the ankle to the end range of eversion (ICC, 0.82–0.96).^[32]^

#### 2.5.2. Berg Balance Scale (BBS)

The Berg Balance Scale (BBS) is a widely used assessment method for evaluating balance in older adults, as it consists of items related to daily activities and can be administered relatively quickly. It comprises 14 items covering sitting, standing, and postural changes, with scores ranging from 0 to 4, yielding a maximum score of 56. The BBS demonstrated high intra-rater (*R* = 0.97) and inter-rater reliability (*R* = 0.99), indicating strong internal consistency in assessing balance abilities.^[33]^

#### 2.5.3. Timed Up and Go test (TUG)

The TUG test is a dynamic balance assessment used to evaluate the risk of falls among older adults. The test consists of the following components: rising from a chair, walking 3 meters, turning, walking back 3 meters, and sitting down again. The time taken (in seconds) to complete these tasks was used to assess balance. The participants sat on a standardized armchair (height, 46 cm; armrest, 65 cm), leaned against the backrest, and positioned their arms on the armrest. Upon receiving the researcher’s starting command “Go,” participants rose from the chair, walked 3 meters at their usual pace, turned around the cone placed at the 3-meter mark, and returned to sit back in the chair. The time required to perform the movements was measured. In community-dwelling older adults, the TUG test demonstrated excellent intra-rater reliability (ICC = 0.99).^[34]^

#### 2.5.4. Gait parameter

Gait analysis was conducted using the FootScan platform system (RSScan International, Olen, Belgium) to determine various parameters related to gait performance. These parameters included step duration (s), step length (mm), step velocity (m/s), step cadence (steps/min), and proportion of double-limb support (%) in the gait cycle. For gait analysis, all participants walked 3 times on a mat at a comfortable pace. An evaluator stood beside each participant during the assessment to guard against sudden loss of balance or falls. A 20-second rest period was provided between each attempt.

#### 2.5.5. Center of pressure (COP) parameter

The trajectory of the center of pressure (COP), commonly known as a stabilogram, during static balance, is frequently used to assess postural control. When standing still, the COP is considered an indicator of the motor mechanisms that maintain balance by keeping the center of mass (COM) within the base of support. Falls are associated with COP displacement at the limits of stability, highlighting the importance of evaluating dynamic balance to assess fall risk.^[35]^ In this study, COP data were obtained using the Footscan platform system. During static balance recording, participants were invited to stand on the platform. Participants then stood in a quiet stance with eyes open, looking straight ahead, arms at their sides, and feet comfortably positioned within the designated area on the platform. In this position, the COP trajectory was recorded for 30 seconds, a duration shown to be sufficient for quantifying postural control using standard variables.^[36]^

### 2.6. Statistical analysis

The general characteristics of the participants were compared using the χ^2^ test. The Wilcoxon signed-rank test/Paired *t*-test was used for comparisons within groups both at baseline and after the interventon. The Wilcoxon Rank-Sum Test/Independent *t*-test was used to compare the mean differences in values between groups before and after the intervention. All statistical analyses were performed using SPSS statistical software (version 26.0; IBM Corp., Armonk), with the significance level set at 0.05.

## 3. Results

### 3.1. Baseline demographics

The general characteristics of the participants are summarized in Table 1. No statistically significant differences were observed between the experimental and control groups in terms of age (*P* = .69), gender distribution (*P* = 1.00), height (*P* = .69), weight (*P* = .91), body mass index (*P* = .80), and foot size (*P* = .11).

**Table 1:** Participants’ general characteristics.*

	Experimental group (N = 22)	Control group (N = 18)	*P*-value
Age (yr)	69.6 ± 2.97	68.2 ± 2.66	.69
Gender			
Male	2 (9.1%)	2 (11.11%)	1.00
Female	20 (90.9%)	16 (88.88%)	
Height (cm)	157.2 ± 5.16	156.5 ± 5.05	.69
Weight (kg)	59.05 ± 11.63	58.08 ± 9.21	.91
BMI (kg/m^2^)	24.78 ± 4.35	24.60 ± 3.51	.80
Foot size (mm)	235.9 ± 8.40	240.0 ± 6.86	.11

BMI = body mass index.

* Values are expressed as mean ± SD.

### 3.2. Ankle joint ROM

The changes in ankle joint ROM after the intervention are presented in Table 2. In the experimental group, ankle dorsiflexion and eversion ROM showed significant improvements compared to pre-intervention measurements (*P* < .001), whereas the control group showed significant improvement only in ankle dorsiflexion ROM (*P* < .001). There was no significant difference in ankle dorsiflexion ROM between the groups (*P* = .07), the change in ankle eversion ROM from pre- to post-intervention was significantly greater in the experimental group compared with the control group (*P* < .001).

**Table 2:** Comparison of ankle range of motion between experimental and control group.*

Variable	Experimental group (N = 22)	Control group (N = 18)	Group diff	*P*-value**
Ankle dorsiflexion ROM				
Pre	18.86 ± 2.68	18.94 ± 2.21	–	–
Post	24.23 ± 2.93	23.5 ± 2.18	–	–
Mean diff	5.37	4.56	0.81	.07
*P*-value***	<.001	<.001	–	–
Ankle eversion ROM				
Pre	14.64 ± 2.72	15.56 ± 3.18	–	–
Post	19.73 ± 2.68	16.72 ± 1.99	–	–
Mean diff	5.09	1.17	3.92	<.001
*P*-value***	<.001	.06	–	–

ROM = range of motion.

* Values expressed as mean ± SD.

** Significance at *P*-value <.05 (intergroup comparison using Independent *t* test or Wilcoxon Rank-Sum Test).

*** Significance at *P*-value <.05 (within groups comparison using Paired *t* test or Wilcoxon signed-rank test).

### 3.3. Balance ability

The changes in balance ability following the intervention are presented in Table 3.

**Table 3:** Comparison of balance ability between experimental and control group.*

Variable	Experimental group (N = 22)	Control group (N = 18)	Group diff	*P*-value**
BBS (score)				
Pre	54.50 ± 1.79	54.17 ± 1.50	–	–
Post	55.09 ± 1.11	54.72 ± 1.13	–	–
Mean diff	0.59	0.55	0.04	.40
*P*-value***	.04	.01	–	–
COP range ML (mm)				
Pre	10.27 ± 4.87	10.78 ± 8.68	–	–
Post	8.18 ± 4.18	10.22 ± 6.99	–	–
Mean diff	−2.09	−0.56	1.53	.04
*P*-value***	.02	.93	–	–
COP range AP (mm)				
Pre	20.73 ± 8.92	18.89 ± 14.88	–	–
Post	20.32 ± 8.58	16.89 ± 8.32	–	–
Mean diff	−0.41	−2.0	1.59	.43
*P*-value***	.93	.96	–	–
COP Velocity (mm/s)	–	–	–	–
Pre	11.15 ± 6.57	10.81 ± 4.54	–	–
Post	9.05 ± 4.15	9.43 ± 2.86	–	–
Mean diff	−2.1	−1.38	0.72	.49
*P*-value***	.03	.04	–	–

AP = anteroposterior, BBS = Berg Balance Scale, COP = center of pressure, ML = mediolateral.

* Values expressed as mean ± SD.

** Significance at *P*-value <.05 (intergroup comparison using independent *t* test or Wilcoxon Rank-Sum Test).

*** Significance at *P*-value <.05 (within groups comparison using paired *t* test or Wilcoxon signed-rank test).

#### 3.3.1. Berg Balance Score (BBS)

Both the experimental and control groups showed significant improvements in BBS scores from pre- to post-intervention (experimental group: *P* = .04; control group: *P* = .01). However, there was no significant difference between the experimental and control groups in the change in BBS scores from pre- to post-intervention (*P* = .40).

#### 3.3.2. COP range

The COP range showed a significant decrease only in the mediolateral (ML) direction in the experimental group from pre- to post-intervention (*P* = .02), whereas non-significant decreases were observed in the anteroposterior (AP) direction in the experimental group (*P* = .93) and in both AP and ML direction in the control group (AP direction: *P* = .96; ML direction: *P* = .93). The change in COP range from pre- to post-intervention was significantly greater in the experimental group compared with the control group, specifically in the ML direction (*P* = .04).

#### 3.3.3. COP mean velocity

Changes in static balance ability, as measured by the mean velocity of the COP, showed significant improvement in both the experimental and control groups after the intervention compared to pre-intervention (experimental group: *P* = .03; control group: *P* = .04). However, there was no significant difference between the groups in the change in COP mean velocity from pre- to post-intervention (*P* = .49).

### 3.4. Gait performance

The changes in gait ability following the intervention are presented in Table 4.

**Table 4:** Comparison of gait performance between experimental and control group.*

Variable	Experimental group (N = 22)	Control group (N = 18)	Group diff	*P*-value**
Step length (mm) (L→R)				
Pre	484.23 ± 76.55	488.61 ± 89.14	–	–
Post	512.14 ± 60.36	499.50 ± 48.02	–	–
Mean diff	27.91	10.89	17.02	.47
*P*-value***	.04	.59	–	–
Step length (mm) (R→L)				
Pre	469.05 ± 74.71	472.44 ± 63.68	–	–
Post	484.95 ± 62.38	495.80 ± 36.86	–	–
Mean diff	15.91	23.34	7.43	.72
*P*-value***	.28	.15	–	–
Step duration (s) (L→R)				
Pre	0.562 ± 0.07	0.607 ± 0.08	–	–
Post	0.534 ± 0.04	0.594 ± 0.07	–	–
Mean diff	−0.028	−0.013	0.015	.51
*P*-value***	.08	.38	–	–
Step duration (s) (R→L)				
Pre	0.554 ± 0.06	0.604 ± 0.07	–	–
Post	0.547 ± 0.06	0.582 ± 0.06	–	–
Mean diff	−0.007	−0.022	0.015	.52
*P*-value***	.57	.11	–	–
Step velocity (m/s) (L→R)				
Pre	3.16 ± 0.68	2.84 ± 0.55	–	–
Post	3.48 ± 0.56	3.06 ± 0.41	–	–
Mean diff	0.32	0.22	0.10	.60
*P*-value***	.04	.06	–	–
Step velocity (m/s) (R→L)				
Pre	3.14 ± 0.69	2.84 ± 0.47	–	–
Post	3.43 ± 0.53	3.10 ± 0.36	–	–
Mean diff	0.29	0.26	0.03	.82
*P*-value***	.03	.04	–	–
Step cadence (step/min)				
Pre	108.41 ± 11.75	99.61 ± 10.34	–	–
Post	113.59 ± 8.43	104.70 ± 8.30	–	–
Mean diff	5.18	5.06	0.12	.97
*P*-value***	.04	.03	–	–
Double support proportion (%)				
Pre	32.88 ± 4.39	31.90 ± 3.29	–	–
Post	30.45 ± 3.10	30.69 ± 2.98	–	–
Mean diff	−2.43	−1.21	1.22	.22
*P*-value***	.01	.04	–	–
Timed Up and Go test (s)				
Pre	9.02 ± 1.37	9.75 ± 1.99	–	–
Post	8.21 ± 1.11	9.36 ± 1.57	–	–
Mean diff	−0.81	−0.4	0.41	.03
*P*-value***	<.001	.13		

* Values expressed as mean ± SD.

** Significance at *P*-value < .05 (intergroup comparison using independent *t* test or Wilcoxon Rank-Sum Test).

*** Significance at *P*-value < .05 (within groups comparison using paired *t*-test or Wilcoxon signed-rank test).

#### 3.4.1. Step length

In the experimental group, step length from left to right (L→R) significantly increased after the intervention (*P* = .04), whereas the change in right-to-left (R→L) step length was not statistically significant (*P* = .28). In the control group, step length increased on both sides, but these changes were not significant (L→R: *P* = .59; R→L: *P* = .15). No significant between-group differences were observed in step length changes from pre- to post-intervention (L→R: *P* = .47; R→L: *P* = .72).

#### 3.4.2. Step duration

Step duration on both sides decreased in both the experimental and control groups; however, these changes were not statistically significant (L→R: *P* = .08 and.38, respectively; R→L: *P* = .57 and.11, respectively). No significant between-group differences were observed in step duration changes from pre- to post-intervention (L→R: *P* = .51; R→L: *P* = .52).

#### 3.4.3. Step velocity

Step velocities on both sides significantly increased in the experimental group (L→R: *P* = .04; R→L: *P* = .03). In the control group, only the right-to-left step velocity showed a significant improvement (*P* = .04), while the change in the left-to-right direction was not significant (*P* = .06). No significant between-group differences were found in step velocity changes (L→R: *P* = .60; R→L: *P* = .82).

#### 3.4.4. Step cadence

Both the experimental and control groups showed significant improvements in step cadence from pre- to post-intervention (*P* = .04 and *P* = .03, respectively), with no significant between-group difference observed (*P* = .97).

#### 3.4.5. Double limb support proportion in gait cycle

Both the experimental and control groups demonstrated a significant decrease in double support proportion during the gait cycle (*P* = .01 and *P* = .04, respectively), but there was no significant difference in the amount of change between the groups (*P* = .22).

#### 3.4.6. Timed Up and Go (TUG) test

The experimental group showed significant decrease in the TUG test performance from pre- to post-intervention (*P* < .001), whereas the control group exhibited a non-significant decrease (*P* = .13). A comparison of the pre- to post-intervention changes revealed significantly greater improvement in the experimental group than in the control group (*P* = .03).

## 4. Discussion

This study aimed to investigate changes in balance and gait abilities in healthy older adults by providing interventions consisting of isolated plantar flexor stretching through “Wall stretching” and simultaneous stretching of the plantar flexors and invertors using a motorized stretching board “Ankle Stretcher. ” In this study, both the experimental and control groups showed significant improvements in static and dynamic balance abilities and gait performance following each intervention. These results demonstrate a significant association between ankle ROM and gait ability in older adults.

Stretching exercises induce biomechanical and physiological changes within the muscles, which can alter muscle activation, strength, and sensory perception. Additionally, they cause viscoelastic changes in the muscles and tendons, leading to increased muscle length and joint ROM.^[37,38]^ These factors may influence postural control and balance. Supporting this, several studies, including Martinez-Jimenez et al,^[14]^ have reported improvements in balance following stretching exercises.^[16,39]^

The COP Range is a representative measure of postural control that evaluates postural equilibrium in both the anterior-posterior (A/P) and medial-lateral (M/L) directions. An increase in the COP Range indicates decreased postural control, whereas a decrease suggests increased postural stability.^[40]^ Laughton et al^[41]^ reported that elderly fallers exhibit significantly greater sway in the A/P direction during static standing. In addition, individuals with lower clinical balance assessment scores tended to show greater postural sway and increased muscle activity in both the A/P and M/L directions. Bok et al^[6]^ observed increased A/P and M/L sway in older adults, with A/P sway associated with decreased plantar flexor strength and M/L sway associated with decreased ankle eversion ROM. Furthermore, in a systematic review, Quijoux et al^[42]^ reported a difference in the M/L-direction COP range between older fallers and non-fallers. These findings suggest that, with increasing age, greater AP and ML sway are required to maintain static balance. In this study, a significant reduction in the COP range in the M/L direction was observed only in the experimental group after the intervention. In addition, between-group comparisons revealed that the experimental group showed significantly greater improvement than the control group in the M/L direction. These results suggest that ankle joint stretching exercises positively affect static balance ability and that the reduction in ML sway may be associated with increased flexibility of the subtalar joint. Consistent with previous studies, the findings of this study suggest that the relationship between aging and the maintenance of static balance, particularly concerning ML sway, is associated with ankle eversion ROM.

Mean COP velocity, which reflects the total distance traveled by the COP over time, is widely regarded as a normalized indicator of sway length and is commonly used to assess the effectiveness of exercise interventions.^[43]^ An increase in mean COP velocity during the upright stance is believed to indicate a decrease in the ability to control posture, whereas a decrease suggests an increase in the ability to control and maintain posture.^[40,44]^ Mean COP velocity is influenced by age-related postural changes and may contribute to the prediction of falls.^[45]^ Previous studies have found that COP velocity is significantly correlated with age-related neuromuscular phenomena, such as increased co-contraction strategies of agonist and antagonist muscles in the lower limbs.^[46,47]^ Sun et al^[48]^ suggested that the perception of COP velocity might be an important factor influencing the control of ankle extensor activity through anticipatory strategies. In this study, both the experimental and control groups showed a significant decrease in mean COP velocity post-intervention. These findings indicate that ankle joint stretching exercises led to changes in the co-contraction patterns of the agonist and antagonist muscles, resulting in improved muscle coordination and an enhanced postural control during upright stance. Although no statistically significant differences were observed between the groups after the intervention, the experimental group demonstrated a greater reduction in the mean COP velocity. These results suggest that, as discussed earlier regarding the COP range, the reduction in ML directional sway due to increased subtalar joint flexibility contributed to a decrease in total COP traveled distance over time, resulting in a larger reduction in COP velocity in the experimental group.

In a study by Alizadehsaravi et al,^[49]^ older adults showed a decrease in step-width variability during narrow-base and normal gaits after 10 sessions of standing balance training. During the single-support phase of the normal gait, a certain degree of control over the center of mass is required,^[50]^ and the reduced step-width variability observed in both conditions reflects a transition toward this control. Variability in foot placement during gait is associated with fall risk^[51,52]^; thus, a decrease in step-width variability can be interpreted as a positive outcome. These findings suggest that standing balance training improves gait balance skills and thereby enhances gait stability. Similarly, in our study, both the experimental and control groups showed improvements in static standing balance through a decrease in the COP travel distance and COP range post-intervention. Additionally, the experimental group showed significant improvements in step length (L→R), step velocity, and step cadence, while the control group showed significant improvements in step velocity (R→L) and step cadence post-intervention. Our results suggest that balance improvements in older adults may translate into beneficial gait outcomes.

Double-limb support acts as a stabilizing factor in the normal gait cycle, and changes in double-limb support with age have been observed. As their dynamic balance control declines relative to young adults, older adults compensate by reducing step length while walking the same number of steps. This adjustment leads to increased time spent in the double-limb-support phase and reduced time in the single-limb-support phase, thereby contributing to a more stable gait pattern. However, this increased stability leads to decreased walking speed, thereby impairing walking efficiency.^[53,54]^ In our study, both the experimental and control groups showed a significant decrease in the double-limb support phase during post-intervention gait. These results suggest that ankle joint stretching exercises have a positive effect not only on static balance but also on dynamic balance control.

As mentioned earlier, following the intervention, the experimental group showed significant improvements in step length (L→R), step velocity, and step cadence, while the control group exhibited significant improvements in step velocity (R→L) and step cadence. However, a significant improvement in the TUG test was observed only in the experimental group after the intervention. A comparison of change scores between the groups also revealed a significant difference, favoring the experimental group over the control group. These results are likely associated not only with improvements in postural control but also with changes in the viscoelastic properties of the muscle-tendon unit. Ankle pronation corresponds to the early stance phase of gait, whereas supination marks the initiation of the propulsion phase when the heel begins to lift off the ground. Dorsiflexion and eversion are the primary pronation components. Immediately after the heel strike during the gait cycle, rapid eversion of the calcaneus begins and continues through mid-stance. Hopkins et al^[55]^ reported that muscle activation of the ankle evertors is approximately 20 to 40% greater during the early stance and toe-off phases of the gait cycle. It is well established that activated muscles exhibit improved performance during the concentric phase when stretched before contraction. Many previous studies have suggested that this phenomenon results from the strain energy stored in tendon structures.^[56–58]^ Combined with previous findings, stretching exercises appear to enhance the viscoelastic properties of tendon structures, thereby increasing the elastic energy stored during the stretch-contracting cycle. Similarly, in the experimental group in this study, increased dorsiflexion and eversion mobility led to greater stretching of the plantar flexors and tibialis posterior muscles during pronation during walking. These biomechanical changes likely enhanced the elastic energy stored in the muscle-tendon units, thereby facilitating a more effective toe-off and generating greater forward propulsive force. TUG test performance is highly dependent on gait speed. The significant improvement in TUG test performance observed in the experimental group compared to the control group, along with a non-significant yet greater increase in step velocity, may be attributed to increased propulsive force.

Previous research has demonstrated a close association between gait speed, stride length, double-limb support time, frequency of falls, and fear of falling.^[51]^ A study comparing the gait patterns of older fallers and non-fallers found that older fallers walked slower with shorter steps and exhibited longer stance phases due to an increase in double-limb-support time.^[59]^ The TUG test is commonly used as a screening tool to identify patients at risk of falling. A previous systemic review reported a mean difference of 0.63 seconds (95% confidence interval = 0.14–1.12, *P* = .01) between fallers and non-fallers among healthy older adults.^[60]^ In our study, both the experimental and control groups showed significant increases in step velocity and cadence after the intervention, along with a significant reduction in the proportion of double-limb support during gait. In the TUG test, the experimental group showed a significant decrease of 0.81 seconds after intervention, whereas the control group showed a non-significant decrease of 0.4 seconds. Based on these results, ankle stretching exercises may reduce the risk of falls in older adults. These findings suggest that improvements in ankle dorsiflexion ROM alone may be effective; however, based on a systematic review of the TUG test,^[60]^ a combined increase in both ankle dorsiflexion and eversion ROM may have a greater impact on reducing fall risk in older adults. However, given the remaining uncertainty regarding the clinical significance of the mean difference in TUG test results between healthy older adult fallers and non-fallers, our findings should be interpreted with caution.

In this study, we compared the effects of simultaneous stretching exercises targeting both the ankle plantar flexors and inverters (experimental group) with those targeting only the ankle plantar flexors (control group). The findings of this study confirmed that ankle joint stretching exercises can improve gait function in older adults and may contribute to fall prevention. These functional improvements are presumed to result from enhanced postural control and changes in viscoelastic properties of the muscle-tendon units. Both the experimental and control groups showed statistically significant improvements in static and dynamic balance as well as gait performance; however, variations were observed across individual outcome measures. Interestingly, the experimental group demonstrated greater average improvements across most outcome measures compared to the control group. We believe that the observed trend may be attributable to changes in ankle eversion ROM. Menz et al^[61]^ reported a significant association between ankle dorsiflexion ROM and toe plantar flexion strength, balance, and functional ability. Previous studies have reported that the ankle eversion ROM plays a crucial role in determining balance and functional abilities in older adults.^[5,6]^ Horak et al^[62]^ reported that the initial use of the ankle strategy in response to external forces requires adequate joint ROM and ankle muscle strength. Considering these findings, it is not surprising that the experimental group, having shown increased ankle dorsiflexion and eversion ROM, demonstrated greater changes in balance and gait outcomes.

Various forms of rehabilitation therapy have been performed, including strengthening and stretching, to improve balance ability in previous studies.^[7–12]^ Mecagni et al^[4]^ suggested that incorporating ankle joint ROM enhancement programs into balance exercise programs is effective in reducing falls and improving balance in older women. Based on our findings, we propose that balance exercise programs for older adults, and stretching exercises that can increase not only dorsiflexion ROM but also eversion ROM should be included.

Our study has several limitations that must be addressed. First, the small sample size limited the generalizability of the study results. Second, the study involved a limited population. We conducted the study in healthy older adults without severe comorbidities, and approximately 90% of the participants were women. Although this predominance reflects demographic patterns commonly observed in community-based rehabilitation studies,^[63]^ it also partly resulted from recruitment circumstances, as many of the volunteers were hospital caregivers, who in our setting are predominantly women. This imbalance may limit the applicability of our results to older men, and the findings should therefore be generalized with caution. Restricting analyses to women only would have substantially reduced statistical power and compromised the randomized design; therefore, the full dataset was retained. Consequently, balance measurements may also have been more susceptible to ceiling effects, and the results may vary across groups with different age and sex distributions. Third, we did not compare different stretching durations. Since both the experimental and control groups underwent prolonged stretching interventions, muscle responses may have approached anatomical limits, potentially underestimating between-group differences in balance and gait improvements. Fourth, evaluations were performed by non-blinded evaluators, which may have introduced bias. Finally, the study lacked long-term follow-up to assess the sustainability of the intervention effects. Therefore, the clinical significance of the results should be interpreted with caution. Future studies should address these limitations by evaluating the effects of ankle stretching exercises on balance and gait in older adults under various intervention conditions using larger and more balanced samples. In particular, future trials would benefit from stratifying analyses by sex or specifically enrolling sufficient numbers of both men and women, as well as conducting larger multicenter studies to enhance the generalizability of findings.

## 5. Conclusion

Stretching of the ankle plantar flexor alone and combined stretching of the plantar flexors and invertors in 40 healthy older adults showed promising improvements in static and dynamic balance as well as gait function. Improvements in postural control and changes in the viscoelastic properties of the muscle-tendon unit likely contributed to enhanced gait function in older adults. These effects were more pronounced with simultaneous stretching of the plantar flexor and inverter muscles. Therefore, ankle stretching exercises should be included in optimal exercise programs to enhance balance and prevent falls among older adults. Specifically, such interventions should target improvements in both dorsiflexion ROM and eversion ROM.

## Acknowledgments

We express our deep gratitude to all participants and the researchers who performed the measurement and analysis of the experiment in the study.

## Author contributions

**Conceptualization:** Soo-Kyung Bok.

**Data curation:** Hyeon Woo Ryoo.

**Formal analysis:** Hyeon Woo Ryoo.

**Funding acquisition:** Soo-Kyung Bok.

**Investigation:** Hyeon Woo Ryoo, Myeong Hyeon Cho.

**Methodology:** Hyeon Woo Ryoo.

**Project administration:** Soo-Kyung Bok.

**Resources:** Soo-Kyung Bok.

**Supervision:** Soo-Kyung Bok.

**Validation:** So Young Ahn.

**Visualization:** Hyeon Woo Ryoo.

**Writing – original draft:** Hyeon Woo Ryoo.

**Writing – review & editing:** Soo-Kyung Bok, So Young Ahn.
